# An Analysis of the Correlation between the Asymmetry of Different EEG-Sensor Locations in Diverse Frequency Bands and Short-Term Subjective Well-Being Changes

**DOI:** 10.3390/brainsci14030267

**Published:** 2024-03-11

**Authors:** Betty Wutzl, Kenji Leibnitz, Masayuki Murata

**Affiliations:** 1 Graduate School of Information Science and Technology, Osaka University, Suita 565-0871, Osaka, Japan; murata@ist.osaka-u.ac.jp; 2 Center for Information and Neural Networks (CiNet), National Institute of Information and Communications Technology, Suita 565-0871, Osaka, Japan; leibnitz@nict.go.jp

**Keywords:** electroencephalography, EEG, brain-signal asymmetry, lateralization, subjective well-being, SWB, environmental conditions

## Abstract

We focus on finding a correlation between the asymmetries of electroencephalography (EEG) signals and subjective well-being (SWB) when changed on short time scales via environmental conditions. Most research in this field focuses on frontal alpha asymmetry. We systematically examine different sensor locations and filter the sensor data into the delta band, the theta band, the alpha band, the beta band, and the gamma band, or leave the EEG signal unfiltered. We confirm that frontal alpha asymmetry is correlated to SWB. However, asymmetries between other sensors and/or filtering the data to other bands also shows a linear correlation to SWB values. Asymmetries of anterior brain regions show statistically significant results not only in the alpha band but also in the delta band and theta band, or when the data is not filtered into a specific band. Asymmetries of posterior regions show a trend to be correlated to SWB when EEG activity is higher on the opposite hemisphere and filtered into different frequency bands. Thus, our results let us conclude that focusing just on frontal sensors and the alpha band might not reveal the whole picture of brain regions and frequency bands involved in SWB.

## 1. Introduction

Since its introduction in 1929 [1], human electroencephalography (EEG) has become a very important tool in clinical applications and research. EEG recordings are often analyzed in different frequency bands. As discussed, for example, in [2], the specific frequency interval chosen for each frequency band varies from study to study. We use the following intervals for the frequency bands: the delta band (0.5–3 Hz), the theta band (4–7 Hz), the alpha band (8–13 Hz), the beta band (14–30 Hz), and the gamma band (>31 Hz). Despite the fact that the limits of those frequency bands are not always defined in exactly the same way and that there are also different sub-bands of interest, each of the above-mentioned frequency bands seems to stem from activities originating in different areas of the brain and also plays an important role in different brain functions. The lowest frequency band, i.e., the delta band, is important for motivational processes, and the theta band plays a significant role in memory and emotional regulation [3]. Alpha oscillation, on the other hand, seems to serve inhibitory processes [3]. The beta band supposedly signals the status quo for movement as well as cognition [4]. The highest frequency band, namely, the gamma band, increases when any sort of change or attention is required [4]. Even though those frequency bands seem to have different functions, they also influence each other and are not independent from each other, e.g., the alpha band is reportedly influenced by other frequency bands, such as the theta band or the beta band [5,6].

At first sight, the brain might seem symmetrical in many ways, e.g., in shape, dynamics, and communication processes. However, it is already known that it is actually asymmetric; see [7,8,9]. One method to measure those asymmetries, which we will also use in this paper, is EEG. Such an asymmetry measured by EEG was already reported in the early years of EEG research, e.g., [10,11]. The most studied EEG asymmetry is by far the frontal alpha asymmetry (FAA). This asymmetry was shown to be internally reliable and quite stable over time [12,13,14,15]. A more recent paper with a large cohort of participants [16] concluded that the less discussed parietal EEG alpha asymmetry is even stabler than the frontal EEG alpha asymmetry. Ocklenburg et al. [17] investigated asymmetry in different frequency bands and locations. They found the highest significant hemispheric asymmetry in the alpha band but also in the theta band, and the beta band showed significant hemispheric asymmetry while the delta band failed to reveal such an asymmetry. Focusing on the sensors of the EEG, they found a positive asymmetry, representing a greater power on the right hemisphere than on the left for the alpha band over the electrode sites C3/C4, P7/P8, and also FC3/FC4. The electrode sites C3/C4 also showed a positive asymmetry for the theta band and the beta band. Some other combinations of sites and frequency bands showed strong trends but could not reach statistical significance. The most investigated combination of F3/F4 asymmetry in the alpha band failed to reach statistical significance in this study by Ocklenburg et al. When focusing on regions of interest rather than on specific electrodes, they reported a significantly rightward asymmetry for central and parieto-occipital areas in the alpha band and a leftward asymmetry for frontal areas in the delta band. The authors also pointed out that the asymmetries of data filtered into different frequency bands are not independent of each other. They found that the average asymmetries of data filtered into the alpha band correlate with those of the delta band, the theta band, and the beta band. Moreover, results from the beta band were correlated with those from the delta band, which again showed a correlation to the theta band [17]. The previously mentioned study [16] replicated the results for the parietal alpha asymmetry but did not report the same results for the frontal sensors. Thus, the authors concluded that the specific EEG system and sensor locations have an influence on the results and recommended including multiple electrode pairs in alpha asymmetry analyses [16].

Besides a general asymmetry in the brain, asymmetries have also been shown to be associated with different mental-related or emotion-related states. The correlation between FAA and mental health has been the subject of most of the literature in this field; see [18] for a review. Some examples include the following: higher FAA was associated with higher self-reported subjective well-being (SWB) [19] or with lower depression and anxiety [20]. However, there are also studies that contradict those results. Generally, there is a huge heterogeneity in the study designs and data processing. Even though the correlation between FAA and depression has gained a lot of attention, van der Vinne et al. concluded in their review that FAA cannot be seen as a reliable diagnostic biomarker for major depressive disorder [21]. FAA is said to reflect approach and avoidance impulses and seems to be involved in top-down control when regulating emotions; see, for example, [22,23]. For further information on this topic, we refer to the meta-analysis in [24] or the cross-sectional study in [25].

Davidson discussed already a while ago the importance of distinguishing between anterior and posterior differences when focusing on hemispheric activation [26]. However, up to today, the differences and correlations between anterior and posterior regions are not fully understood. Henriques and Davidson reported a difference in alpha asymmetry over frontal but also posterior regions as a marker for depression [27]. Those findings were supported by newer research, e.g., [28,29]. Such a parietal alpha asymmetry was also associated with posttraumatic stress disorder (PTSD) [30], the risk for major depression disorder [31], or attentional bias to threat [32]. Moreover, asymmetries in frequency bands other than the alpha band have also been reported to correlate to mental conditions. EEG beta asymmetry was found to be correlated to attention deficit hyperactivity disorder (ADHD) [33,34] and, when focused on frontal areas, could be used to predict trait aggressive tendencies and behavioral inhibition [35]. Asymmetries of parieto-occipital areas in the alpha band and the beta band were correlated to the hedonic valuation of food [36]. Asymmetries have also been related to sleep stages; namely, asymmetries of the first sleep stage were found to be decreased over fronto-central areas in the delta band, the theta band, the upper alpha band, and all three defined sub-bands of the beta band, whereas an increase was observed in parieto-occipital areas in the theta band, the two alpha sub-bands, and in two out of three beta sub-bands [37]. A study from 2021 included over 300 participants and found no association between multidimensional well-being and FAA. However, they reported a correlation in the temporo-parietal areas with lower left than right activity, corresponding to higher well-being levels. For the delta band, the theta band, or the beta band, they did not find any correlation between asymmetries and well-being [38].

In this paper, we will focus on SWB and its correlation to EEG asymmetries. Different terms for similar conditions have been used. Those include for example “subjective happiness” [39], “psychological well-being” [40], or “quality of life” [41]. It is also closely related to “comfort”; see [42] for a discussion about differences and similarities between some of those terms. There is a relationship between well-being and the brain; however there, is still a lot that is currently unknown [43]. Many studies use questionnaires, such as the five-item World Health Organization Well-Being Index (WHO-5), to determine SWB [44]. We will use an expanded Likert scale [45] for determining SWB on a scale from 1 to 10, and details can be found in Section 2.1.

In our previous work, we could show that a correlation between FAA and SWB also holds for short-time scales [46]. This expands previous knowledge, which described this correlation on larger time scales and when psychological or psychiatric interventions were performed. We now extend our previous work and see if we can find a correlation between the asymmetries of different electrode sites as well as different frequency bands to such a short-term SWB. As discussed above, the role of asymmetries in frequency bands other than the alpha band is not often reported in the literature. Furthermore, a majority of the studies in this field focus on frontal areas. Hence, we want to shed some light into less researched frequency bands and sensor locations.

The three main research questions we aim to answer in this work are the following:

(RQ1) Is there a correlation between short-term SWB and EEG asymmetries filtered in frequency bands other than the alpha band?

(RQ2) Do the sensor locations influence those results, especially anterior versus posterior sensor asymmetry?

(RQ3) Does re-referencing the EEG signals change the results?

## 2. Materials and Methods

### 2.1. Experiment

The experiment was conducted in 2022 and first analyses of this data with the focus on FAA are already published in [46]. Before starting the experiment, we obtained approval of the local ethics committee at Osaka University. We recruited 30 students from Osaka University (28 right-handed, 2 left-handed, 16 males, 14 females, ages 22.3 ± 4.2 years). The goal of the experiment was to change SWB on a short time scale by changing the environmental conditions. The experimental room was located at Suita Campus of Osaka University. We divided the room with partitions into an area of 1.4 m × 2.1 m. A desk, a chair, shelves with temperature–humidity sensors, heating devices, humidifiers and dehumidifiers were placed within this area. The participant came to the experimental site and, after an explanation of the experiment in either Japanese or English, signed a written consent form. An EEG headset (EPOC+, EMOTIV, San Francisco, CA, USA) was placed on the participant. The participants were sitting on the chair at the desk during the runs and were asked to rate their SWB on a scale from 1 to 10 every 30 s. A SWB value of 10 should represent the most comfortable feeling with the physical environment in terms of temperature–humidity and overall well-being. A SWB value of 1 should stand for the least comfort and make the participant want to leave the situation immediately. The experimenter reminded the participants every 30 s by saying the word ‘number’ to state their SWB during each EEG recording. We recorded EEG data for up to 9 min for each of the six different temperature–humidity settings in the room, namely, low, middle, and high temperatures in combination with low and high humidities. The specific values, as measured with temperature–humidity sensors of [47], can be found in the Supplementary Material of [46]. After finishing a run, the participant could relax and the experimenter changed the temperature and humidity of the room with off-the-shelf equipment to the next temperature–humidity setting. This was repeated until either all 6 runs were performed or the scheduled time for the session was over.

### 2.2. Asymmetry Calculation

The EEG headset used for the experiment is equipped with 14 electrodes, namely, AF3, F7, F3, FC5, T7, P7, O1, O2, P8, T8, FC6, F4, F8, and AF4. The layout follows the standard 10–20 system. The common mode sense (CMS) is located at P3 and the driven right leg (DRL) at P4. This device operates using sequential sampling with a single analog-to-digital converter and an internal sampling rate of 2048 Hz which can be down-sampled to either 256 or 128 Hz. We chose the down-sampling rate of 128 Hz in our experiment. The recording bandwidth was 0.16–43 Hz and a digital notch filter at 50 and 60 Hz was applied automatically.

For the EEG preprocessing, we wrote a MATLAB (R2022a) [48] pipeline using EEGLAB (v2022.0) [49] following HAPPE (The Harvard Automated Processing Pipeline for Electroencephalography) [50], making use of MARA (Multiple Artifact Rejection Algorithm) [51,52]. We refer the interested reader to our last paper [46] for all details about the EEG preprocessing steps. After loading the EEG data as EDF (European Data Format) files and disabling non-EEG channel recordings, we identified the sensors locations by their names in the 10–20 system. The next step was detrending, followed by performing bad channel detections based on probability, also performed twice as in [50]. After a wavelet-ICA (independent component analysis), our pipeline used MARA, which automatically flags and rejects artifact components of EEG data [51,52]. MARA has been shown to be very effective when it comes to removing muscle artifact components, see, e.g., [50]. Hence, no other steps to eliminate such artifacts were performed.

After applying the EEG preprocessing pipeline, we chose specific time intervals for the asymmetry calculation. In our previous work, when we focused on FAA, we found that 10 s up to the time when SWB was reported by the participants was the ideal interval for the asymmetry calculation. Hence, we used those same time intervals here. After choosing two channels, $ch_{1}$ and $ch_{2}$, and making sure that they were not marked as bad channels during the preprocessing step, the time series of the specific interval was filtered in the frequency interval of interest, i.e., delta from 0.5 to 3 Hz, theta from 4 to 7 Hz, alpha from 8 to 13 Hz, beta from 14 to 30 Hz, gamma from 31 to 100 Hz, or ‘non’ from 0.5 to 100 Hz. The upper bound of 100 Hz for the gamma band and the non band was just arbitrarily selected because the algorithm needs an upper limit. The EEG headset records on a bandwidth between 0.16 and 43 Hz; therefore, the actual upper limit for our recordings was 43 Hz. We also included the non band in our analyses to be sure that differences in results were not due to the computational filtering step but indeed from a specific frequency band. Then, we followed the descriptions given in [53,54] and computed the Fast Fourier Transform with a 50% overlapping Hanning window. The power density of the chosen frequency band was calculated for $ch_{1}$ and $ch_{2}$ in each chosen interval, i.e., from 10 s before up to the time the SWB was reported. The asymmetry Asym was then determined by subtracting of the natural logarithm of the power density of $ch_{1}$ filtered into the specific frequency band, denoted as band in the formula, minus the natural logarithm of the power density of $ch_{2}$ filtered into band via the formula

(1) $$Asym_{band}\left(ch_{1},ch_{2}\right)=mean\left(\log pow_{band}\left(ch_{1}\right)-\log pow_{band}\left(ch_{2}\right)\right),$$

with log being the natural logarithm, $pow_{band}(ch_{1})$ the power spectrum of the signal on the first channel $ch_{1}$, and $pow_{band}(ch_{2})$ being the power spectrum of the second channel $ch_{2}$, both filtered into the specific frequency band, denoted as band.

### 2.3. Re-Referencing the Data

**Figure 1:**
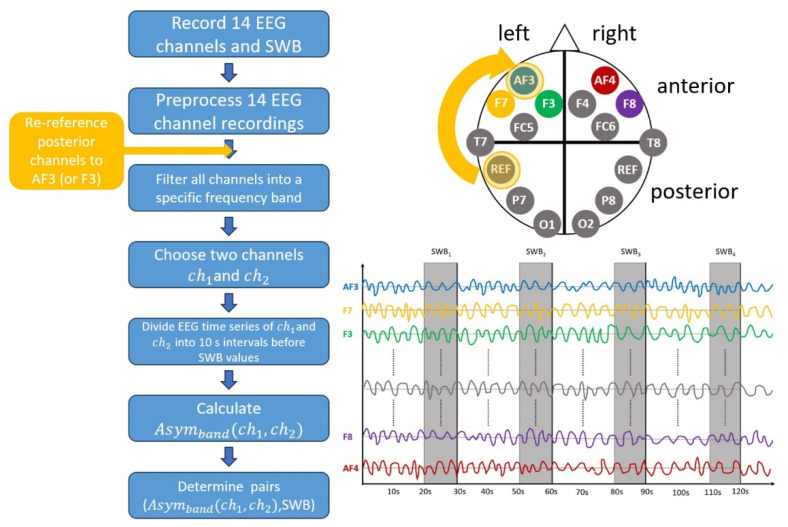
Representation of our workflow on the left, channel locations and time series used for Asym calculation on the right.

Making the right choice for the reference electrode is very challenging; see [55] for a discussion. The popular Cz reference was reported to under- or overestimate activity and showed lower correlation to other reference schemes [56,57]. The current-source density (CSD) transformation was successfully used in several papers, e.g., [58,59,60]. One caveat of this method is that it does a poor job on sparse EEG headsets. For example, the authors of [55] state that a number of 60 channels works well for the CSD transformation. Thus, such a transformation is not suitable for our EEG headset. The reference electrode should be located as far away as possible from the EEG sensors of interest to obtain reliable results. The posterior sensors, especially T7 and P7, are located closely to the reference electrode, which might yield invalid asymmetries. Thus, the same analysis was performed for all posterior sensors, i.e., T7, P7, O1, O2, P8, and T8 in our setup, but with an additional re-referencing step to either AF3 or F3. One might argue that especially AF3 might be influenced by ocular signals. Although such signals should have been eliminated during the preprocessing step, we chose two different new reference electrodes for comparison. An overview of the workflow from the recorded EEG and SWB values to the final $Asym_{band}(ch_{1},\;ch_{2})$ pairs is shown on the left in Figure 1. The yellow part represents the optional re-referencing step for the posterior sensors. Moreover, the EEG sensor layout and the time intervals used for the Asym calculations are shown on the right of Figure 1.

### 2.4. Averaging over Different Sensors

Another issue when it comes to calculating EEG asymmetries, especially FAA, is that there is no consensus in the literature on which specific EEG sensors to use. In our previous work [46], we chose AF3 and AF4 for the calculation of FAA. However, F7 and F8 are also popular choices. To eliminate the choice of two specific EEG sensors, we divided the brain into quadrants, i.e., right and left anterior as well as right and left posterior. The sensors in the specific quadrants were averaged, i.e., AF3, F3, F7, and FC5 for the left anterior quadrant, AF4, F4, F8, and FC6 for the right anterior quadrant, T7, P7, and O1 for the left posterior quadrant, and T8, P8, and O2 for the right posterior quadrant. Those averaged signals were then used as $ch_{1}$ and $ch_{2}$ in Equation (1) for the calculation of Asym.

### 2.5. Correlation between Asym and SWB

Now we have Asym corresponding to the SWB for each interval of one participant, the next step is to determine a relationship between those two values. In [46], we showed that there is a positive linear correlation between $Asym_{alpha}(AF4,\;AF3)$ and SWB. Here, we want to test whether there are combinations, other than AF4/AF3 and the alpha band, that also give a linear correlation between the asymmetry and SWB. Despite using different frequency bands and different channels for the calculation, we encounter the same problem that we had when trying to find a correlation between FAA and SWB in [46]; namely, we have an imbalanced data set. Most participants reported SWB values of 6, 7, or 8 more frequently than SWB values of 1, 2, 3, or 10. Performing a linear regression without accounting for that imbalance would lead to overfitting the more frequently reported values and underfitting the less frequently reported values. Hence, we used SMOTE (Synthetic Minority Over-Sampling Technique) as described in [61] and implemented in imbalanced-learn (0.10.1) [62] using python (3.9.18) [63] and scikit-learn (1.1.3) [64]. This technique creates synthetic data points that follow the same empirical distribution as the original data. Before applying SMOTE, we first checked whether each SWB value given by the individual participant was reported at least 3 times, which is the minimum number of data points needed for the method to run. If one SWB value was given less than 3 times, this specific SWB value and its Asym values were deleted. Then, we also excluded data from participants if they ended up with less than 3 different SWB values. This was done because with less than 3 SWB values, a linear regression is not reasonable. After applying SMOTE to the remaining data, we ended up with a balanced amount of (Asym, SWB) pairs per given SWB value. Then, a linear regression was performed using the method in [64]. Since SMOTE gives slightly different results each time, it was repeated 10 times per participant. The 10 slopes and intercepts per individual participant were then averaged. Finally, we tested for a linear relationship using a two-sided t-test to see if the slope is different from zero and determined the *p*-value and the 95% confidence interval (CI) via the method in scipy (1.12.0) [65].

### 2.6. Overall Statistical Analysis

The algorithm described above was run for all combinations of sensors and bands. Since we performed a lot of statistical tests, we must assume that some of the statistically significant results are actually Type 1 errors (false positives). If many outcomes are tested for statistical significance, some may appear statistically significant with a *p*-value smaller than 0.05 simply by chance and not because of a true underlying significance. One possible solution for such a multiple comparison problem is the Bonferroni correction. However, this correction is very conservative and also increases the risk of introducing Type 2 errors (false negatives). So, we decided not to use the Bonferroni correction but instead focus on the false discovery rate (FDR), which controls the proportion of errors made when rejecting hypotheses and is described in [66]. More specifically, we used the expansion from independent or positively correlated tests to generally dependent tests from [67], as implemented in statsmodels (0.14.0) [68]. We decided to choose an alpha of 0.1, i.e., 10% of our results are expected to be false positives, because we wanted to prioritize sensitivity over specificity.

## 3. Results

In this section, we will focus on the most significant results. Since it is not fully clear how handedness affects asymmetries, see [16,17] for a discussion. We excluded the two left-handed participants from our analyses. Another participant reported less than three different SWB scores and, thus, was also excluded.

### 3.1. Results with the Original Reference Electrode

**Figure 2:**
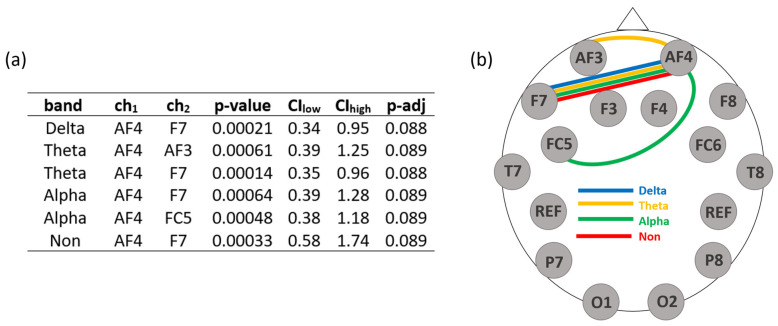
Statistically significant results of the analyses with the original reference system. (**a**) The first column shows the considered frequency band. Entries in the second and third columns give the EEG channels used for $ch_{1}$ and $ch_{2}$ in Equation (1). The fourth column shows the *p*-value of the two-sided t-test. The fifth and six columns give the lower and upper bound of the CI of the slope of the linear correlation, respectively. The remaining column gives the adjusted *p*-value as calculated via the Benjamini–Yekutieli FDR procedure. (**b**) The figure provides a graphical representation of the findings. Different line colors represent different frequency bands.

First, we present the results using the original reference electrode. We analyzed asymmetries between all contralateral sensors in the anterior and all contralateral sensors in the posterior regions. We did not calculate asymmetries between frontal and posterior contralateral sensors because of the general anterior–posterior difference, which was already reported in the literature a while ago [26]. Moreover, in order to compare our results to the FAA literature, we choose the $ch_{1}$ from the right hemisphere and $ch_{2}$ from the left hemisphere. The alpha in the Benjamini–Yekutieli FDR procedure was set to 0.1. Correlations which turned out to be statistically significant are listed in Figure 2. Each channel combination in Figure 2 includes the channel AF4. The combination of $ch_{1}=AF4$ and $ch_{2}=F7$ is of special interest, as it proves to be statistically significant for each frequency band except the beta band and the gamma band.

Figure 2 does not include any posterior sensors. To investigate the relationship of posterior sensors further, we now focus on just the posterior region, i.e., sensors T7, P7, O1, T8, P8, and O2. In Table 1, we list the results of Asym from the posterior pairs for which the analysis reaches an unadjusted *p*-value under 0.1. We see that no asymmetry calculation is listed for EEG data in the delta band or the alpha band. Furthermore, either the lower or the upper bound of the CI is always close to 0.

### 3.2. Results after Re-Referencing the EEG Data

The next results we present are those from analyses after re-referencing the EEG data. We start with the results for AF3 as the new reference sensor. The signals at the posterior sensors (T7, P7, O1, O2, P8, and T8) were re-referenced to the frontal sensor AF3 while the rest of the analyses remained the same. Table 2 shows the results of the analyses that yielded an unadjusted *p*-value of less than 0.1 Comparing this table to Table 1, we see that after re-referencing, more tests yielded an unadjusted *p*-value smaller than 0.1. Moreover, results can be found in all frequency bands considered. However, Table 2 can just be seen as trend results since none of the results are statistically significant after performing the Benjamini–Yekutieli FDR procedure.

Then, we performed the same analysis but re-referenced the posterior sensors to the frontal sensor F3; see Table 3. Compared to Table 1, we obtain again more channel pairs. Analogous to Table 2, results in all frequency bands appear. Table 2 and Table 3 are similar but not identical, and we want to point out that also the entries in Table 3 can just be seen as trends since no *p*-value passed the statistical significance after the Benjamini–Yekutieli FDR procedure with an alpha equal to 0.1.

### 3.3. Results from Signals Averaged over Brain Quadrants

Finally, we focused on averaging the signals from the left anterior quadrant, i.e., AF3, F3, F7, and FC5, and the right anterior quadrant, i.e., AF4, F4, F8, and FC6, with the original reference electrode, and also averaged the electrodes on the left posterior quadrant (T7, P7 and O1) and the right posterior quadrant (T8, P8, O2) for both new reference electrodes, i.e., AF3 and F3; see Section 2.4. Those averaged signals were then again correlated to the corresponding SWB value, followed by a statistical analysis. Table 4 shows the results from these analyses. Statistically significant results with *p* < 0.1 can be found in all bands except the delta and gamma bands. There are results for the asymmetries of anterior and posterior quadrants; however, again, none of those pass statistical significance via the Benjamini–Yekutieli FDR procedure with an alpha equal to 0.1. Looking at Table 4, we see a trend for a reversed direction, i.e., for anterior quadrants, the correlation slope of the linear correlation is positive, whereas for the posterior regions it shows a negative trend. When it comes to the posterior quadrants, it is worth noticing that only results with data re-referenced to F3 showed an unadjusted *p*-value under 0.1. Neither the original data nor re-referencing to AF3 could show any trends.

## 4. Discussion

We systematically analyzed the correlation between the asymmetries of pairs of individual EEG sensors and averaged over brain quadrants when filtering the EEG signal into different frequency bands. The literature mostly focuses on FAA, which we also studied in our previous work [46]. In that work, we showed that FAA is correlated to short-term SWB. Here, we went a step further and analyzed whether such a correlation also holds for asymmetry values calculated from other sensors than AF4 and AF3 and/or when filtering into different frequency bands. We specifically did not predefine a direction of the asymmetry, i.e., we checked for more activity in the right hemisphere compared to the left but also vice versa. Moreover, we included asymmetries from all sensors to each other, not just from hemispheric counterparts. This was done for two reasons. First, we wanted to make sure that asymmetries do not just appear for different sensors in the EEG headset, and second, we used a headset with a low number of sensors and fixed sensor locations, so it was not possible to adjust for head shape. When setting up the experiment, we tried to find the best fit of the EEG headset, but because of those shortcomings and the fact that we did not check the specific sensor location using MRI, we analyzed all sensor combinations so as not to miss any results that might be due to an inaccurate sensor location.

### 4.1. Discussion of the Results with the Original Reference Electrode

The first result, presented in Section 3.1 and Figure 2, shows a statistically significant positive linear correlation between Asym and the short-term SWB for different sensor combinations and frequency bands. Hence, we can answer (RQ1) with the following: *Yes, there is a correlation between short-term SWB and EEG asymmetries in frequency bands other than the alpha band.* Among all the sensor pairs whose Asym shows a statistically significant correlation to SWB, the sensor with the higher power is located on the right hemisphere. All statistically significant results are from the Asym of AF4 combined with other sensors in the frontal areas. Of specific interest is the combination of AF4 with F7, i.e., Asym(AF4, F7), which has a positive linear correlation to SWB in any frequency band except the beta band and the gamma band. As those sensors are located in the frontal area, our results replicate the results in the literature: however, the two sensors are not each other’s exact hemispherical counterparts. We cannot comment on the importance of this finding because we did not confirm the exact location of the sensors. Hence, it is not entirely clear how accurately we can talk about the specific underlying brain areas. To our knowledge, there is no paper that discusses the specific meaning of an asymmetry between one brain region and another which is located slightly next to its hemispheric counterpart. The main conclusion here is that brain activity is lateralized and, when correlated to short-term SWB, a higher activity on the right than on the left is observed in frontal areas in different frequency bands.

Next, we turn to the comparison of anterior results to posterior results. In Figure 2, no combination of posterior sensors appears. We answer (RQ2) with the following: *Yes, the sensor locations do influence the results*. We could not find statistically significant results for sensors in the posterior brain region. In order to further investigate this topic, we looked at unadjusted *p*-values to see if there was any trend visible. Table 1 gives the results of the analyses with an unadjusted *p*-value of 0.1. Although they are not statistically significant, the asymmetries of some posterior sensors show a trend towards having a linear correlation with short-term SWB. What is interesting here is that trends can be found for all considered frequency bands except the delta band and the alpha band.

### 4.2. Discussion of Results after Re-Referencing the EEG Data

As discussed in Section 2.3, re-referencing EEG data to a different reference electrode was reported to influence asymmetries. Thus, we performed the same analyses as before but limited to the sensors T7, P7, O1, O2, P8, and T8 and added a re-referencing step in the analyses. We chose two different new reference electrodes, AF3 and F3. The results with the re-reference electrode AF3 are shown in Table 2. These results again should be considered only as trends since they are not statistically significant after the Benjamini–Yekutieli FDR procedure with an alpha equal to 0.1, and the stated *p*-values are unadjusted. We see that after adding a re-referencing step, we find trends for all considered frequency bands. Similar but not identical trends were found when using F3 as the new reference electrode; see Table 3. Again, this table shows that unadjusted *p*-values and are not statistically significant after the Benjamini–Yekutieli FDR procedure with an alpha equal 0.1. The combination of O2 and P7 seems to be of specific interest in the posterior region because their Asym filtered into different frequency bands indicates a trend to be linearly correlated to short-term SWB. This leads us to the following answer (RQ3): *Yes, re-referencing the EEG signal changes the results.* In this way, re-referencing makes the trend towards statistical significance stronger; however, in our case, it does not reach statistical significance. This is in alignment with the literature that already discussed the influence of the reference electrode on asymmetries (see Section 2.3).

We can conclude that there is a trend towards a linear correlation between the EEG asymmetry of posterior regions and short-term SWB, which hints towards a lateralization in the posterior regions. It is important to point out that the asymmetry of the posterior regions is in the opposite direction to that of the anterior regions. In other words, the slope of this trend is negative. This means that, here, less power of a specific frequency band on the right is correlated to short-term SWB. We find again that the sensor location of the seemingly most important combination of O2 and P7 are not their exact hemispheric counterparts, but sensors are located close to them. The reasons for that are probably similar to those for the anterior areas, which we discussed in Section 4.1.

### 4.3. Discussion of Results from Signals Averaged over Quadrants

Since it is not fully clear to which extent the exact sensor location influences the results, we performed some analyses, where we averaged EEG signals from all sensors in the left anterior quadrant (AF3, F3, F7, and FC5) and all sensors located in the right anterior quadrant (AF4, F4, F8, and FC6). Then, we analyzed whether Asym from the EEG signal averaged over those quadrants yielded any statistically significant results when correlated to SWB. Analogous analyses were performed with averaged signals from all sensors in the left posterior quadrant (T7, P7, and O1) and right posterior quadrant (T8, P8, and O2) for different reference electrodes. The results (*p* < 0.1 unadjusted) can be seen in Table 4. First, we see that by filtering into different bands yields trends for the comparison of different quadrants, i.e., for the correlation between Asym and SWB of anterior quadrants, we find trends for the theta band and the alpha band, whereas for the posterior quadrants the beta band shows a trend towards a linear correlation. Not filtering the EEG signal (non band) leads to a correlation trend between the Asym from both the anterior and posterior quadrants and short-term SWB. Filtering in the delta band or gamma band did not give any trends. When comparing the results from anterior and posterior quadrants, we see that the direction of the asymmetry is again reversed. More power of the specific frequency band in the right anterior quadrant than in the left anterior quadrant resulted in a correlation to SWB, whereas more power in the left posterior quadrant than in the right posterior quadrant correlated to SWB. The unadjusted *p*-value for analyses from the anterior quadrants are lower and the CI bounds are farer away from 0, which makes those results more valuable than the results for the posterior quadrants. However, again, none of the results show statistical significance after the Benjamini–Yekutieli FDR procedure with an alpha equal to 0.1 and thus just show trends. Another point to keep in mind is that just re-referencing the EEG signal to F3 yielded trends for posterior quadrants, whereas re-referencing for AF3 or keeping the original reference electrode did not. This again points to the importance of the reference electrode when it comes to asymmetries.

When considering the different frequency bands, we notice that analyses including anterior quadrants show a trend towards statistical significance in the theta band and the alpha band. This is not surprising since FAA is the most researched among the EEG asymmetries, and, as we could also show in our previous paper [46], FAA is correlated to short-term SWB. So, finding a correlation between the $Asym_{alpha}$ of the averaged EEG signal from all frontal sensors and not just AF4 and AF3 to SWB was expected, especially because there is no consensus in the literature on which two frontal sensors to use when calculating FAA, and results using different sensors from the frontal areas were reported. The correlation between EEG asymmetries when filtered into the theta band is less reported. Our study points towards the correlation between asymmetries of anterior sensors filtered in the theta band being equally significant to those filtered in the alpha band when correlating those values to short-term SWB. The beta band seems to be important when it comes to asymmetries in the posterior quadrants. Reports about a correlation between beta asymmetries and mental states are rarely found but were reported to correlate with ADHD and the valuation of food [33,34,36]. We conclude that the asymmetries of posterior quadrants when filtered in the beta band might also be suitable for measuring SWB, at least when SWB is varied on a short-term basis.

### 4.4. General Discussion

We also analyzed all our results without filtering into any frequency band, referred to as the non band. We did this because frequency bands, while having some neuroscientific meaning, are used very inconsistently throughout the literature. During our analyses, the non band often resulted in statistically significant results. This might be due to the fact that the most important frequency band is the dominant one and thus also dominates the asymmetry calculation without specific filtering. This result might also be of interest when considering low cost and fast EEG preprocessing. Moreover, the specific sensor location might not even be that important, but the region is. We hypothesize that our results would also hold for just one sensor in the anterior right and one sensor in the anterior left side, or analogous for the posterior regions. If such an EEG system proved to be successful, it might find an application in smart-home environments where it could be used to control the environment (e.g., air conditioner, light, aroma, etc.) to change a person’s SWB in the physical environment [69,70]. Knowing that filtering the signals is not necessary to find a correlation between EEG asymmetries and short-time SWB might be useful to save computational power and time in such a smart-home application.

### 4.5. Limitations of the Study

Finally, we want to mention some of the limitations of our study and the results presented in this paper. First, we want to point out that our study focuses on short-term SWB. We evaluated SWB every 30 s for a maximum of 9 min. Since this is a very short time interval, we cannot conclude that our results will also hold for long term SWB. In order to capture the whole extent of the correlation between EEG asymmetries and SWB, our findings would also have to be evaluated on longer time scales.

We did not discuss any neurobiological explanations for the observed correlation between EEG asymmetry and short-term SWB. The literature on the neurobiology of well-being already exists, e.g., [71], but further research has to be conducted to see how such mechanisms hold for short-term changes of SWB.

We discussed the fact that the reference electrode has an effect on asymmetry. Thus, we advise the reader to always keep that in mind when focusing on EEG asymmetry research or when comparing results of studies with different EEG reference electrodes.

Another part that must be kept in mind when thinking about the generalization of our study is our sample of participants. Our participants were all university students and were mainly right-handed. We analyzed EEG data from 15 male and 13 female participants, excluding two left-handed participants, which makes our data set not fully balanced in gender. However, the difference is not significant. Therefore, we assume that any possible bias introduced by imbalanced genders would be negligible. Taken together, our results are limited to our participants and might not be representative of the whole population. Additional studies need to be conducted to clarify whether our results differ with attributes such as age, handedness, education, nationality, etc. Also, a bigger sample size would increase the generalizability and increase the statistical power. However, our study gives some insights into possible correlations. Those need to be tested on a larger scale in the future to guarantee generalizability and also to see whether the trends observed in this paper show statistical significance on a larger data set.

Such a large and extensive study could also lead to more insights into the causal relationship between Asym and SWB. From our study, we can conclude that there is a relationship between these two variables but cannot say anything about their causal relationship.

Short-term SWB is, as indicated by the name, a very subjective measure. We tried to explain to the participants as best as possible what to focus on; however, we cannot exclude the fact that every participant slightly interprets SWB differently. The Likert scale itself has also been criticized, see, e.g., [72]. However, it was the only measure available to be performed on short time scales. Besides that, longer questionnaires are prone to different biases.

In our experiment, we chose temperature and humidity as two environmental factors to influence short-term SWB. However, those are not the only environmental conditions that are expected to influence SWB. Hence, also, different light settings, noise levels, or even different smells might influence one’s SWB. Since our experimental time was limited, we just chose those two variables, but that does not mean that temperature and humidity are superior or more influential than other environmental settings.

## 5. Conclusions

In this work, we analyzed EEG asymmetries between different sensor locations or brain areas as well as including less-investigated frequency bands and their correlation to short-term changes in SWB. First, we can confirm that there is a positive linear correlation between FAA and SWB. We also found that some asymmetries of frontal sensors correlate to SWB when filtered into the delta band or theta band. For posterior regions, an opposite direction of the asymmetry was observed, and the beta band showed, although not statistically significant, higher importance than for the frontal sensors. This makes us conclude that a focus on FAA might exclude insights into brain function. Moreover, not filtering into any frequency band also showed statistically significant results. This might be useful when developing wearable systems that require results with little computational power and in very short times.

## Figures and Tables

**Table 1:** Results of the analyses with the original reference electrode for sensor combinations in the posterior regions and unadjusted *p* < 0.1: The first column shows the considered frequency band. Entries in the second and third columns give the EEG channels used for $ch_{1}$ and $ch_{2}$ in Equation (1). The fourth column shows the unadjusted *p*-value of the two-sided *t*-test. The last two columns give the lower and upper bounds of the CI of the slope of the linear correlation, respectively.

Band	*ch*_1_	*ch*_2_	*p*-Value	CI_low_	CI_high_
Theta	T8	P7	0.067	−0.47	0.02
Theta	P8	O1	0.063	−0.72	0.02
Beta	O2	P7	0.054	−0.95	0.01
Gamma	O2	P7	0.042	−1.05	−0.02
Gamma	T8	O1	0.046	0.01	1.43
Non	O2	P7	0.031	−1.25	−0.07
Non	T8	O1	0.052	−0.00	1.05

**Table 2:** Results of the analyses with AF3 as reference electrode for sensor combinations in the posterior regions and unadjusted *p* < 0.1: The first column shows the considered frequency band. Entries in the second and third columns give the EEG channels used for $ch_{1}$ and $ch_{2}$ in Equation (1). The fourth column shows the unadjusted *p*-value of the two-sided *t*-test. The last two columns give the lower and upper bounds of the CI of the slope of the linear correlation, respectively.

Band	*ch*_1_	*ch*_2_	*p*-Value	CI_low_	CI_high_
Delta	O2	T7	0.048	−0.55	−0.00
Theta	P8	P7	0.096	−0.66	0.06
Theta	P8	O1	0.026	−0.62	−0.04
Alpha	O2	P7	0.011	−0.93	−0.13
Alpha	T8	P7	0.026	−0.71	−0.05
Beta	T8	T7	0.094	−1.08	0.09
Beta	O2	P7	0.014	−1.52	−0.19
Gamma	O2	T7	0.078	−0.92	0.05
Gamma	O2	P7	0.047	−1.30	−0.01
Non	O2	T7	0.056	−1.10	0.02
Non	O2	P7	0.024	−1.84	−0.14

**Table 3:** Results of the analyses with F3 as reference electrode for sensor combinations in the posterior regions and unadjusted *p* < 0.1: the first column shows the considered frequency band. Entries in the second and third columns give the EEG channels used for $ch_{1}$ and $ch_{2}$ in Equation (1). The fourth column shows the unadjusted *p*-value of the two-sided *t*-test. The last two columns give the lower and upper bounds of the CI of the slope of the linear correlation, respectively.

Band	*ch1*	*ch2*	*p*-Value	CI_low_	CI_high_
Delta	O2	T7	0.033	−0.54	−0.02
Delta	O2	P7	0.046	−0.64	−0.01
Delta	P8	P7	0.060	−0.57	0.01
Delta	P8	O1	0.079	−0.52	0.03
Theta	P8	O1	0.048	−0.62	−0.00
Alpha	O2	P7	0.050	−0.86	0.00
Beta	O2	P7	0.009	−1.54	−0.25
Gamma	O2	T7	0.053	−1.58	0.01
Non	O2	T7	0.094	−1.15	0.10
Non	O2	P7	0.023	−2.12	−0.17

**Table 4:** Results of the analyses from averaged quadrants with unadjusted *p* < 0.1: the first column shows the considered frequency band. The second column gives the location of the quadrants, i.e., anterior or posterior. The next column shows the re-referenced electrode for the analysis. This is followed by columns showing the unadjusted *p*-value and the lower and upper bounds of the CI of the two-sided *t*-test.

Band	Area	Re-Ref	*p*-Value	CI_low_	CI_high_
Theta	anterior	-	0.016	0.15	1.33
Alpha	anterior	-	0.019	0.16	1.64
Non	anterior	-	0.058	−0.05	2.51
Beta	posterior	F3	0.073	−1.24	0.06
Non	posterior	F3	0.074	−2.26	0.11

## Data Availability

The datasets generated during and/or analyzed during the current study, as well as all source codes, are available from the corresponding author on reasonable request.
